# Blood urea nitrogen to serum albumin ratio is associated with all-cause mortality in patients with AKI: a cohort study

**DOI:** 10.3389/fnut.2024.1353956

**Published:** 2024-02-20

**Authors:** Yue Shi, Hangyu Duan, Jing Liu, Xiujie Shi, Yifan Zhang, Qi Zhang, Mingming Zhao, Yu Zhang

**Affiliations:** ^1^Department of Nephrology, Xiyuan Hospital, China Academy of Chinese Medical Sciences, Beijing, China; ^2^Beijing University of Chinese Medicine, Beijing, China

**Keywords:** blood urea nitrogen to serum albumin ratio, BAR, acute kidney injury, AKI, all-cause mortality, MIMIC-IV database, cohort study

## Abstract

**Background:**

This study aims to investigate the relationship between blood urea nitrogen to serum albumin ratio (BAR) and all-cause mortality in patients with acute kidney injury (AKI) and evaluate the effect of BAR on the prognosis of AKI.

**Methods:**

Adult patients with AKI admitted to the ICU in the Medical Information Mart for Intensive Care IV (MIMIC-IV) were selected in a retrospective cohort study. BAR (mg/g) was calculated using initial blood urea nitrogen (mg/dl)/serum albumin (g/dl). According to the BAR, these patients were divided into quartiles (Q1–Q4). Kaplan–Meier analysis was used to compare the mortality of the above four groups. Multivariate Cox regression analysis was used to evaluate the association between BAR and 28-day mortality and 365-day mortality. The receiver operating characteristic (ROC) curve was plotted and the area under the curve (AUC) was calculated, and the subgroup analysis was finally stratified by relevant covariates.

**Results:**

A total of 12,125 patients with AKI were included in this study. The 28-day and 365-day mortality rates were 23.89 and 39.07%, respectively. Kaplan–Meier analysis showed a significant increase in all-cause mortality in patients with high BAR (Log-rank *p* < 0.001). Multivariate Cox regression analysis showed that BAR was an independent risk factor for 28-day mortality (4.32 < BAR≤7.14: HR 1.12, 95% CI 0.97–1.30, *p* = 0.114; 7.14 < BAR≤13.03: HR 1.51, 95% CI 1.31–1.75, *p* < 0.001; BAR>13.03: HR 2.07, 95% CI 1.74–2.47, *p* < 0.001; Reference BAR≤4.32) and 365-day mortality (4.32 < BAR≤7.14: HR 1.22, 95% CI 1.09–1.36, *p* < 0.001; 7.14 < BAR≤13.03: HR 1.63, 95% CI 1.46–1.82, *p* < 0.001; BAR>13.03: HR 2.22, 95% CI 1.93–2.54, *p* < 0.001; Reference BAR ≤ 4.32) in patients with AKI. The AUC of BAR for predicting 28-day mortality and 365-day mortality was 0.649 and 0.662, respectively, which is better than that of blood urea nitrogen and sequential organ failure assessment. In addition, subgroup analysis showed a stable relationship between BAR and adverse outcomes in patients with AKI.

**Conclusion:**

BAR is significantly associated with increased all-cause mortality in patients with AKI. This finding suggests that BAR may help identify people with AKI at high risk of mortality.

## 1 Introduction

Acute kidney injury (AKI) is a clinical syndrome of rapid renal function decline in a short period of time caused by various diseases and is a common critical disease in clinical practice (1). Approximately 10 to 15% of hospitalized patients suffer from AKI, and the prevalence of AKI even exceeds 50% in intensive care unit (ICU) patients (2, 3). There is still no specific treatment and the mortality rate is high, although the nephrology community is paying increasing attention to AKI. According to the International Society of Nephrology, approximately 1.7 million people die of AKI worldwide annually (4). In addition, AKI is associated with moderate to severe disability, chronic kidney disease (CKD), end-stage renal disease, cardiovascular disease, longer hospital stays, and increased costs (5, 6). Therefore, identifying AKI at high risk of mortality in the ICU is critical to improving its prognosis. Previous studies have shown that certain clinical biomarkers, such as serum creatinine (Scr), cystatin C, and urinary protein, are associated with AKI prognosis (7, 8). However, a recognized biomarker for predicting the prognosis of critical patients with AKI has not been established. It is therefore necessary to investigate suitable risk stratification further to determine the prognosis of critical patients with AKI.

Blood urea nitrogen to serum albumin ratio (BAR) is a novel prognostic biomarker discovered in recent years. Blood urea nitrogen (BUN) is the main metabolite of human protein. BUN is an important parameter reflecting the relationship between renal status, protein metabolism, and nutritional status and will be increased when the glomerular filtration rate is decreased (9). Albumin is also a reflection of the nutritional status of the body. Furthermore, albumin has various physiological characteristics, such as antioxidant and anti-inflammatory (10). BAR combines the nutritional status and renal status of the organism; it is relatively non-invasive and easy to obtain and is an effective tool for predicting the prognosis of critical patients. Previous studies have found that BAR exhibits good mortality prediction ability in numerous diseases including sepsis, pneumonia, chronic obstructive pulmonary disease, and chronic heart failure (11–14). However, the predictive validity of BAR for mortality in AKI has not yet been demonstrated. Therefore, we aimed to investigate the predictive value of BAR for 28-day and 365-day mortality in patients with AKI, thereby providing new clues for the early clinical prediction of AKI.

## 2 Methods

### 2.1 Data sources

In this study, we used a retrospective cohort study to select the publicly available Medical Information Mart for Intensive Care IV (MIMIC-IV) database,^1^ data of ICU hospitalized adults at Beth Israel Deaconess Medical Center between 2008 and 2019. The database is publicly available and database loyalty identifiers are hidden to protect privacy; therefore, this study is exempt from the requirement for informed consent or ethical approval. Data extraction personnel Y. S. participated in training and examinations, obtained permission from the Cooperative Institution Training Program (CITI), and required permission to use the MIMIC-IV database.

### 2.2 Study population

Adult patients with AKI admitted to the ICU for the first time were included. AKI was defined by the Kidney Disease: Improving Global Outcomes (KDIGO) criteria, using both serum creatinine (Scr) and urine output criteria (15). AKI was defined as an increase in Scr level of ≥0.3 mg/dL (≥ 26.5 μmoL/L) within 48 h or an increase in Scr of ≥1.5 times baseline known or presumed to have occurred within the previous 7 days, or urine output of <0.5 mL/kg/h for 6 h. Exclude patients who lack serum albumin and BUN data within 24 h of ICU admission.

### 2.3 Data extraction

Structured Query Language (SQL) running on PostgresSQL (version 13.7.2) was used to extract baseline data 24 h before ICU admission, including sex, age, weight, Sequential Organ failure assessment (SOFA), vital signs including heart rate and blood pressure, respiratory rate, laboratory results, comorbidities, interventions, and other variables. Laboratory parameters included white blood cell (WBC) count, red blood cell (RBC) count, platelet count, hemoglobin, red blood cell distribution width (RDW), Scr, BUN, albumin, blood glucose, serum sodium, and serum potassium, all of which were initial values within 24 h of ICU admission. Comorbidities included CKD, sepsis, hypertension, diabetes mellitus (DM), heart failure, respiratory failure, and malignant tumors. Interventions included renal replacement therapy (RRT), diuretics, and vasoactive drugs. Defined using International Classification of Diseases, 10th Revision (ICD-10) and ICD-9 codes. BAR (mg/g) was calculated as initial BUN (mg/dl)/serum albumin (g/dl).

### 2.4 Management of abnormal and missing values

Variables with outliers were processed by the winsorize method using the STATA winsor2 command with 1 and 99% cutoff points. To address missing values, researchers employed a multiple imputation approach. Variables with a missing value of more than 15% were excluded, such as height, C-reactive protein, lactate, and aminotransferase.

### 2.5 Outcome indicators

The primary outcomes of this study were 28-day mortality and 365-day mortality after AKI diagnosis.

### 2.6 Statistical analysis

Continuous variables were presented as mean ± standard deviation (mean ± SD) for normal distributions and as median (interquartile range) [M (IQR)] for skewed distributions. Comparisons were performed by one-way analysis of variance or the Kruskal-Wallis H test, respectively. Categorical data were presented as frequencies and percentages (%) and compared using the Chi-square test or Fisher’s exact test.

Cox proportional hazards models were used to calculate hazard ratios (HR) and 95% confidence intervals (CI) for BAR and mortality between groups, adjusted for multiple confounding variables through stepwise regression (*p* < 0.05 for selection) (Model 1: unadjusted; Model 2: adjusted for sex, age, and weight; Model 3: adjusted for sex, age, weight, SOFA, Scr, BUN, WBC, Platelet, sepsis, heart failure, respiratory failure, hypertension, DM, and RRT). Variables with variance inflation factors greater than 5 were excluded from the model to prevent multicollinearity. Kaplan–Meier survival analysis was used to assess mortality between BAR-based groups and the Log-Rank test was used to compare the four groups’ curves. A restricted cubic spline (RCS) model was used to investigate the potential dose–response association between BAR and all-cause mortality in patients with AKI. In addition, receiver operating characteristic (ROC) analysis was used to assess the predictive ability of BAR, BUN, and SOFA for 28-day and 365-day mortality, and the area under the curve (AUC) was calculated. Finally, subgroup analysis was used to investigate the consistency of the prognostic value of BAR within different subgroups. These subgroups were based on age; sex; SOFA; complications, such as CKD, sepsis, hypertension, DM, and malignant tumors; and interventions (diuretics and vasoactive drugs). Variance ratio tests assessed interactions between BAR and subgroups. Data analysis was performed using STATA software (Version 16.0) and IBM SPSS software (Version 26.0). Two-tailed tests indicated that *p* < 0.05 was considered statistically significant.

## 3 Results

### 3.1 Baseline characteristics

**Figure 1:**
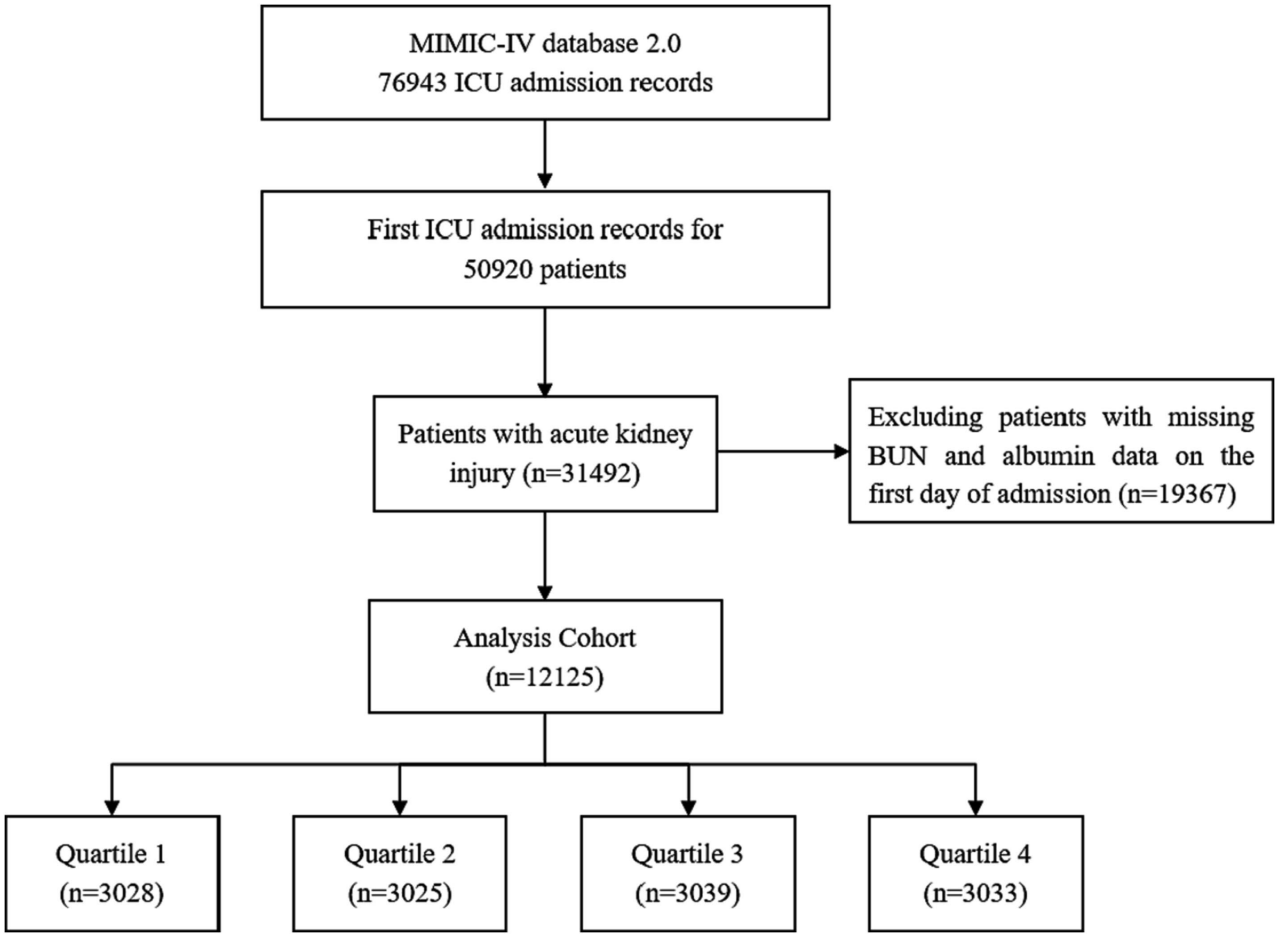
Flow chart of patient selection.

A total of 12,125 patients with an AKI diagnosis were included in this study according to various inclusion and exclusion criteria, and the patient screening flow chart is shown in Figure 1. They were divided into four groups according to quartiles of BAR (Q1: ≤4.32; Q2:4.32–7.14; Q3:7.14–13.03; Q4: >13.03), and the baseline characteristics of the four groups were shown in Table 1. Patients with higher BAR were generally older, had a higher SOFA score, a higher prevalence of CKD, sepsis, DM, heart failure, respiratory failure, and malignant tumors, and a higher use ratio of RRT, diuretics, and vasoactive drugs. RBC, hemoglobin, platelet, and albumin were lower than in the lower BAR group, while WBC, RDW, Scr, BUN, and potassium were higher than in the lower BAR group. With increasing BAR, 28-day mortality (11.26% vs. 17.29% vs. 27.21% vs. 39.80%, *p* < 0.001) and 365-day mortality (19.95% vs. 30.81% vs. 44.03% vs. 57.43%, *p* < 0.001) increased in patients with AKI.

**Table 1:** Baseline characteristics according to BAR quartiles.

Variables	Overall	Q1 (≤4.32)	Q2 (4.32–7.14)	Q3 (7.14–13.03)	Q4 (>13.03)	*p*-value
Participants	12,125	3,028	3,025	3,039	3,033	
Age	66.78 (55.14, 78.55)	58.90 (46.42, 70.22)	67.56 (56.63, 78.91)	70.39 (58.84, 81.39)	70.22 (59.03, 80.96)	<0.001
Male, n (%)	6,933 (57.18)	1,548 (51.12)	1,726 (57.06)	1,791 (58.93)	1,868 (61.59)	<0.001
Weight (kg)	80.00 (67.50, 96.00)	79.40 (66.40, 94.80)	80.00 (67.80, 96.50)	80.00 (67.90, 95.10)	80.90 (68.00, 98.00)	0.002
Vital signs						
HR (beats/min)	86.07 (74.97, 98.81)	85.01 (74.82, 97.19)	85.36 (73.86, 97.90)	86.92 (75.76, 99.80)	87.64 (75.50, 100.79)	<0.001
SBP (mmHg)	114.67 (104.96, 127.62)	119.98 (109.02, 132.78)	115.84 (105.80, 128,41)	112.65 (103.68, 124.96)	110.67 (102.04, 122.25)	<0.001
DBP (mmHg)	62.18 (55.45, 69.88)	66.80 (60.08, 74.98)	63.09 (56.46, 70.68)	60.76 (54.37, 67.62)	58.44 (52.46, 65.37)	<0.001
RR (beats/min)	19.33 (17.00,22.33)	18.58 (16.62, 21.15)	19.17 (16.91, 21.96)	19.85 (17.32, 22.91)	19.96 (17.24, 23.27)	<0.001
SOFA	5 (3, 9)	3 (2, 5)	4 (2, 7)	6 (4, 9)	8 (5, 11)	<0.001
Comorbidities, n (%)						
CKD	2,576 (21.25)	69 (2.28)	346 (11.44)	818 (26.92)	1,343 (44.28)	<0.001
sepsis	7,875 (64.95)	1,533 (50.63)	1,850 (61.16)	2,152 (70.81)	2,340 (77.15)	<0.001
Heart failure	3,486 (28.75)	400 (13.21)	794 (26.25)	1,062 (34.95)	1,230 (40.55)	<0.001
Respiratory failure	4,644 (38.30)	862 (28.47)	1,077 (35.60)	1,255 (41.30)	1,450 (47.81)	<0.001
Hypertension	3,073 (25.34)	812 (26.82)	944 (31.21)	789 (25.96)	528 (17.41)	<0.001
DM	1,848 (15.24)	295 (9.74)	402 (13.29)	516 (16.98)	635 (20.94)	<0.001
Malignant tumors	2,578 (21.26)	458 (15.13)	669 (22.12)	715 (23.54)	736 (24.26)	<0.001
Interventions, n (%)						
RRT	1,415 (11.67)	64 (2.11)	153 (5.06)	402 (13.23)	796 (26.24)	<0.001
Diuretics	3,957 (32.64)	633 (20.90)	1,042 (34.45)	1,156 (38.05)	1,126 (37.11)	<0.001
Vasoactive drugs	3,740 (30.85)	475 (15.69)	805 (26.61)	1,101 (36.24)	1,359 (44.79)	<0.001
Laboratory tests						
RBC (m/μL)	3.55 (3.00, 4.12)	3.88 (3.34, 4.40)	3.69 (3.16, 4.23)	3.44 (2.94, 4.00)	3.21 (2.73, 3.71)	<0.001
WBC (K/μL)	11.00 (7.70, 15.60)	10.10 (7.20, 13.90)	11.00 (7.80, 15.20)	11.20 (7.70, 16.40)	12.00 (8.10, 17.6)	<0.001
Hemoglobin (g/dL)	10.70 (9.00, 12.40)	11.70 (10.10, 13.30)	11.20 (9.60, 12.80)	10.40 (8.90, 11.90)	9.60 (8.30, 11.10)	<0.001
RDW (%)	14.80 (13.60, 16.50)	13.9 (13.10, 15.20)	14.40 (13.40, 15.80)	15.10 (14.00, 16.90)	15.90 (14.60, 17.70)	<0.001
Platelet (K/μL)	186 (125, 252)	204 (149, 264)	187 (132, 250)	177 (118, 243)	167 (106, 246)	<0.001
Scr (mg/dL)	1.10 (0.80, 1.70)	0.70 (0.60, 0.90)	0.90 (0.70, 1.20)	1.30 (1.00, 1.70)	2.10 (1.30, 3.60)	<0.001
BUN (mg/dL)	22 (14, 37)	11 (8, 14)	18 (15, 21)	28 (23, 34)	55 (43, 74)	<0.001
Albumin (g/dL)	3.10 (2.60, 3.60)	3.4 (3.00, 3.80)	3.2 (2.80, 3.60)	3.00 (2.50, 3.40)	2.80 (2.30, 3.20)	<0.001
Glucose (mg/dL)	130 (105, 173)	122 (102, 155)	133 (107, 172)	136 (108, 182)	132 (103, 186)	<0.001
Potassium (mmol/L)	4.10 (3.70, 4.60)	3.90 (3.50, 4.20)	4.10 (3.70, 4.50)	4.20 (3.80, 4.70)	4.50 (3.90, 5.10)	<0.001
Sodium (mmol/L)	138 (136, 140)	138 (137, 140)	138 (136, 140)	138 (136, 140)	138 (135, 139)	<0.001
BAR (mg/g)	7.14 (4.32, 13.03)	3.16 (2.50, 3.75)	5.12 (4.86, 6.30)	9.45 (8.16, 11.07)	19.88 (15.71, 27.07)	<0.001
28-day mortality (%)	2,897 (23.89)	341 (11.26)	523 (17.29)	827 (27.21)	1,206 (39.76)	<0.001
365-day mortality (%)	4,616 (38.07)	604 (19.95)	932 (30.81)	1,338 (44.03)	1,742 (57.43)	<0.001

HR, heart rate; SBP, systolic blood pressure; DBP, diastolic blood pressure; RR, respiratory rate; SOFA, sequential organ failure assessment; CKD, chronic kidney disease; DM, diabetes mellitus; RRT, renal replacement therapy; RBC, red blood cell; WBC, white blood cell; RDW, red blood cell distribution width; SCr, serum creatinine; BUN, Blood urea nitrogen; BAR, Blood urea nitrogen to serum albumin ratio.

### 3.2 All-cause mortality in different groups

**Figure 2:**
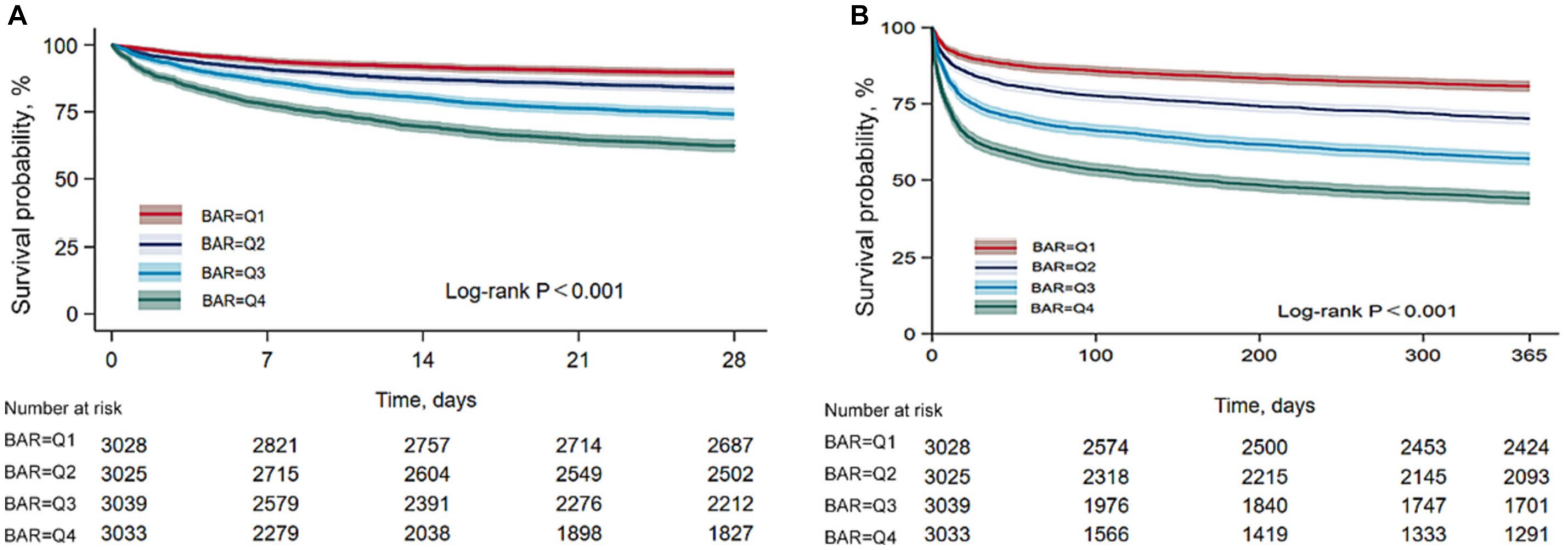
Kaplan–Meier survival analysis curves for all-cause mortality. **(A)** Kaplan–Meier survival analysis curves for 28-day mortality. **(B)** Kaplan–Meier survival analysis curves for 365-day mortality.

A total of 2,897 of 12,125 patients died within 28 days, giving a mortality rate of 23.89%. A total of 4,616 died within 365 days, giving a mortality rate of 38.07%. Kaplan–Meier curves (Figure 2) showed differences in 28-day mortality and 365-day mortality among the four groups, with mortality also increasing progressively as BAR quartiles increased (log-rank *p* < 0.001).

### 3.3 Association between all-cause mortality and BAR

**Figure 3:**
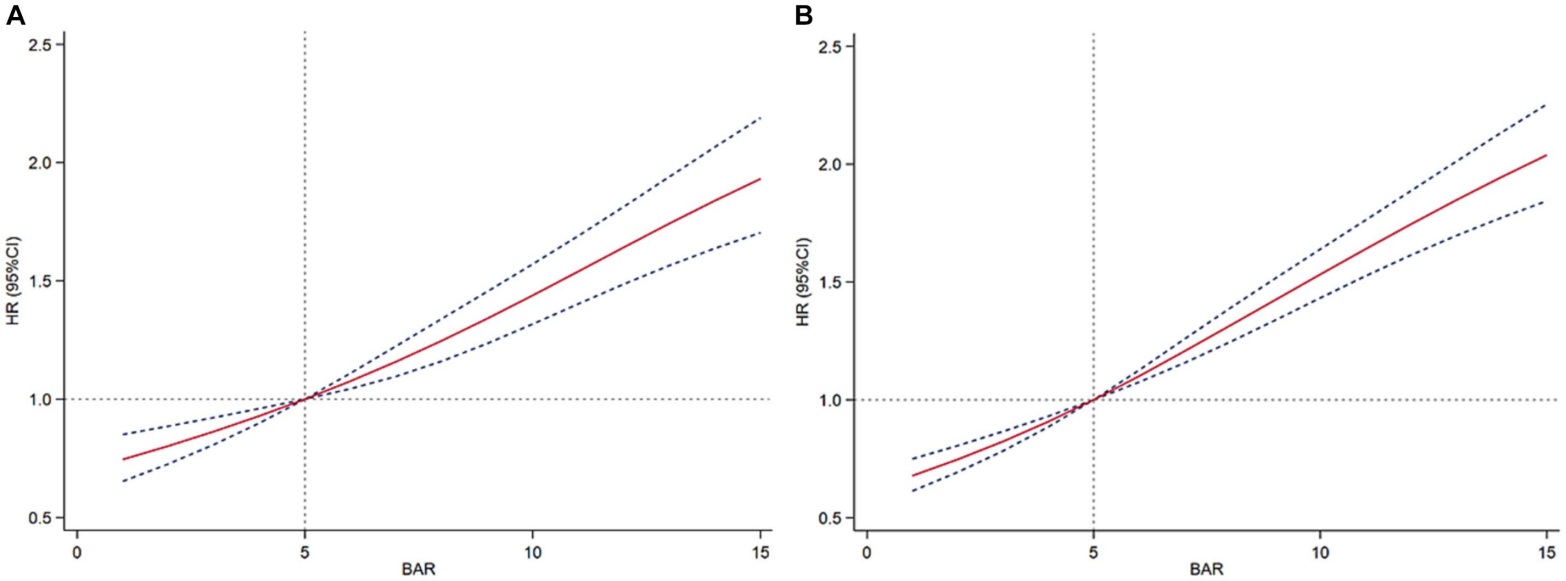
Restricted cubic spline curves of BAR with all-cause mortality. **(A)** Restricted cubic spline for 28-day mortality. **(B)** Restricted cubic spline for 365-day mortality.

We constructed multivariate Cox regression models to assess the independent effect of BAR on 28-day mortality and 365-day mortality in patients with AKI. Cox proportional hazards analysis showed that BAR was significantly associated with 28-day mortality in both the unadjusted model (HR, 1.04 [95% CI 1.03–1.04], *p* < 0.001) and the fully adjusted model (HR, 1.02 [95% CI 1.01–1.03], *p* < 0.001) when BAR was considered as a continuous variable. BAR was also significantly associated with 365-day mortality in the unadjusted model (HR, 1.03 [95% CI 1.03–1.04], *p* < 0.001) and the fully adjusted model (HR, 1.02 [95% CI 1.02–1.03], *p* < 0.001). In addition, when BAR was considered as a nominal variable, the risk of 28-day mortality in the unadjusted model was significantly higher for BAR Q2, Q3, and Q4 than for BAR Q1, with increasing BAR (Q1 vs. Q2: HR, 1.60 [95% CI 1.30–1.84]; Q3: HR, 2.69 [95% CI 2.36–3.07]; Q4: HR, 4.32 [95% CI 3.81–4.90]; *P* for trend <0.001). Similar results were obtained in the Cox proportional hazards analysis of BAR and 365-day mortality (Q1 vs. Q2: HR, 1.65 [95% CI 1.49–1.84]; Q3: HR, 2.63 [95% CI 2.38–2.90]; Q4: HR, 3.94 [95% CI 3.58–4.33]; *P* for trend <0.001). However, the effect of BAR on the risk of death at 28 days and 365 days gradually decreased as the model was adjusted. In fully adjusted models, the 28-day mortality risk was significantly higher for BAR Q3 and Q4 than for BAR Q1 (Q1vs. Q3: HR, 1.51 [95% CI 1.31–1.75]; Q4: HR, 2.07 [95%CI 1.74–2.47]; *P* for trend <0.001), the 365-day mortality risk of BAR Q2, Q3, and Q4 was significantly higher than that of BAR Q1 (Q1vs. Q2: HR, 1.22 [95% CI 1.09–1.36]; Q3: HR, 1.63 [95%CI 1.46–1.82]; Q4: HR, 2.22 [95%CI 1.93–2.54]; *P* for trend <0.001) (Table 2). In addition, RCS regression models showed that a higher BAR (> 5) was associated with an increased risk of death at 28 and 365 days in patients with AKI (Figure 3).

**Table 2:** Cox proportional hazard ratios for all-cause mortality.

Categories	Model 1 HR (95% CI)	*p-*value	Model 2 HR (95% CI)	*p-*value	Model 3 HR (95% CI)	*p-*value
28-day mortality						
BAR	1.04 (1.03–1.04)	<0.001	1.04 (1.03–1.04)	<0.001	1.02 (1.01–1.03)	<0.001
BAR (category)						
Q1 (≤4.32)	Ref.		Ref.		Ref.	
Q2 (4.32–7.14)	1.60 (1.30–1.84)	<0.001	1.46 (1.26–1.68)	<0.001	1.12 (0.97–1.30)	0.114
Q3 (7.14–13.03)	2.69 (2.36–3.07)	<0.001	2.40 (2.10–2.75)	<0.001	1.51 (1.31–1.75)	<0.001
Q4 (>13.03)	4.32 (3.81–4.90)	<0.001	3.92 (3.45–4.46)	<0.001	2.07 (1.74–2.47)	<0.001
*P* for trend		<0.001		<0.001		<0.001
365-day mortality						
BAR	1.03 (1.03–1.04)	<0.001	1.03 (1.03–1.04)	<0.001	1.02 (1.02–1.03)	<0.001
BAR (category)						
Q1 (≤4.32)	Ref.		Ref.		Ref.	
Q2 (4.32–7.14)	1.65 (1.49–1.84)	<0.001	1.44 (1.30–1.61)	<0.001	1.22 (1.09–1.36)	<0.001
Q3 (7.14–13.03)	2.63 (2.38–2.90)	<0.001	2.23 (2.02–2.47)	<0.001	1.63 (1.46–1.82)	<0.001
Q4 (>13.03)	3.94 (3.58–4.33)	<0.001	3.43 (3.11–3.78)	<0.001	2.22 (1.93–2.54)	<0.001
*P* for trend		<0.001		<0.001		<0.001

Model 1 was unadjusted.

Model 2 was adjusted for sex, age, and weight.

Model 3 was adjusted for sex, age, weight, SOFA, Scr, BUN, WBC, Platelet, sepsis, hypertension, heart failure, respiratory failure, DM, and RRT.

### 3.4 Prediction of all-cause mortality in patients with AKI by BAR

**Figure 4:**
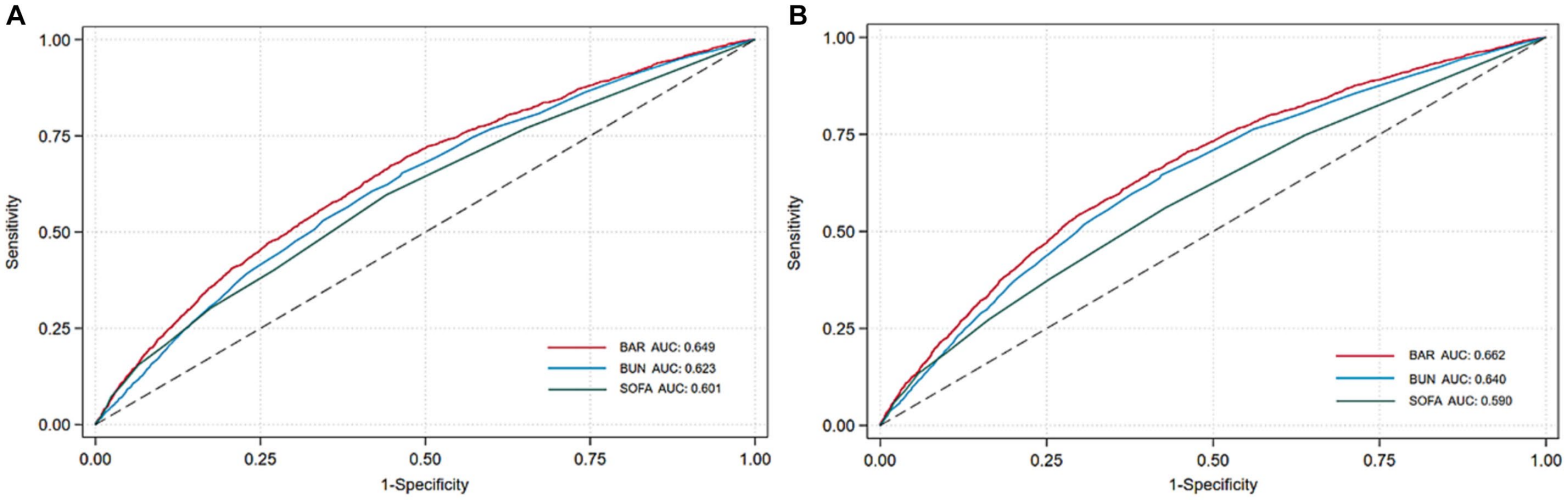
ROC curves of BAR for predicting all-cause mortality. **(A)** ROC curves of BAR for predicting 28-day mortality. **(B)** ROC curves of BAR for predicting 365-day mortality.

We plotted ROC curves for BAR, BUN, and SOFA to assess their predictive value for 28-day and 365-day mortality in patients with AKI. As shown in Figure 4 and Table 3, the AUC of 28-day mortality for BAR was 0.649, which was superior to BUN (AUC = 0.623) and SOFA (AUC = 0.601). The AUC of 365-day mortality for BAR was also better than BUN and SOFA (BAR: AUC = 0.662; BUN: AUC = 0.640; SOFA = 0.590). Therefore, BAR has a good predictive advantage.

**Table 3:** Information of ROC curves.

	AUC (95% CI)	Cutoff value	Sensitivity	Specificity	Accuracy
28-day mortality					
BAR	0.649 (0.636–0.662)	8.07	0.65	0.62	0.63
BUN	0.623 (0.610–0.637)	23.25	0.63	0.59	0.61
SOFA	0.601 (0.587–0.615)	3.50	0.60	0.56	0.58
365-day mortality					
BAR	0.662 (0.659–0.674)	7.14	0.67	0.60	0.64
BUN	0.640 (0.628–0.652)	23.25	0.61	0.63	0.62
SOFA	0.590 (0.578–0.603)	3.50	0.56	0.57	0.57

BAR, Blood urea nitrogen to serum albumin ratio; SOFA, sequential organ failure assessment; BUN, Blood urea nitrogen.

### 3.5 Subgroup analysis

We stratified and interacted on the relationship between BAR and the primary outcome endpoint based on potential confounders such as age, sex, SOFA, CKD, sepsis, hypertension, DM, malignant tumors, diuretics, and vasoactive drugs (Table 4), and the data showed that BAR was robust in predicting 28-day mortality and 365-day mortality. Notably, there was an interaction effect between BAR and sepsis and malignant tumors (*P* for interaction <0.05), and the predictive value of BAR appeared to be more prominent in patients with non-sepsis and non-malignancy.

**Table 4:** Subgroup analysis of the association between all-cause mortality and BAR.

Subgroups	*n*	Q1 (≤4.32)	Q2 (4.32–7.14)	Q3 (7.14–13.03)	Q4 (>13.03)	*p* for trend
28-day mortality						
Age, years						
≤65	5,567	Ref.	1.30 (1.05–1.61)	1.49 (1.19–1.86)	1.82 (1.37–2.42)	<0.001
>65	6,558	Ref.	1.10 (0.90–1.33)	1.66 (1.37–2.01)	2.36 (1.89–2.94)	<0.001
*P* for interaction			0.052	0.500	0.778	
Sex						
Male	6,933	Ref.	1.13 (0.92–1.39)	1.56 (1.27–1.91)	1.90 (1.49–2.42)	<0.001
Female	5,192	Ref.	1.13 (0.92–1.39)	1.49 (1.2–1.83)	2.40 (1.86–3.11)	<0.001
*P* for interaction			0.990	0.397	0.842	
SOFA						
≤5	6,296	Ref.	1.01 (0.82–1.24)	1.40 (1.12–1.77)	1.85 (1.30–2.64)	<0.001
>5	5,829	Ref.	1.26 (1.01–1.56)	1.80 (1.47–2.21)	2.68 (2.14–3.37)	<0.001
*P* for interaction			0.722	0.992	0.438	
CKD						
Yes	2,576	Ref.	1.47 (0.70–3.12)	1.7 (0.84–3.55)	2.78 (1.34–5.77)	<0.001
No	9,549	Ref.	1.12 (0.97–1.31)	1.53 (1.31–1.79)	1.92 (1.57–2.35)	<0.001
*P* for interaction			0.602	0.897	0.653	
Sepsis						
Yes	7,875	Ref.	1.02 (0.86–1.21)	1.34 (1.13–1.57)	1.86 (1.53–2.26)	<0.001
No	4,250	Ref.	1.24 (0.93–1.54)	1.79 (1.34–2.41)	2.31 (1.55–3.44)	<0.001
*P* for interaction			0.018	<0.001	<0.001	
Hypertension						
Yes	3,073	Ref.	0.93 (0.70–1.23)	1.69 (1.28–2.24)	2.50 (1.73–3.61)	<0.001
No	9,052	Ref.	1.21 (1.02–1.43)	1.46 (1.23–1.73)	1.98 (1.62–2.42)	<0.001
*P* for interaction			0.057	0.867	0.980	
DM						
Yes	1,848	Ref.	1.07 (0.63–1.83)	2.28 (1.38–3.75)	2.66 (1.51–4.68)	<0.001
No	10,277	Ref.	1.14 (0.98–1.33)	1.44 (1.24–1.68)	2.01 (1.67–2.42)	<0.001
*P* for interaction			0.957	0.054	0.254	
Malignant tumors						
Yes	2,578	Ref.	1.05 (0.79–1.40)	1.57 (1.17–2.07)	2.23 (1.59–3.11)	<0.001
No	9,547	Ref.	1.10 (0.93–1.31)	1.41 (1.19–1.67)	1.86 (1.51–2.29)	<0.001
*P* for interaction			0.196	0.366	0.850	
Diuretics						
Yes	3,957	Ref.	1.07 (0.80–1.44)	1.70 (1.29–2.25)	2.20 (1.58–3.05)	<0.001
No	8,168	Ref.	1.17 (0.99–1.38)	1.45 (1.22–1.72)	2.02 (1.64–2.49)	<0.001
*P* for interaction			0.476	0.423	0.350	
Vasoactive drugs						
Yes	3,740	Ref.	0.98 (0.77–1.26)	1.29 (1.02–1.63)	1.89 (1.45–2.47)	<0.001
No	8,385	Ref.	1.12 (0.94–1.35)	1.53 (1.27–1.84)	1.93 (1.52–2.46)	<0.001
*P* for interaction			0.201	0.052	0.062	
365-day mortality						
Age, years						
≤65	5,567	Ref.	1.42 (1.21–1.68)	1.73 (1.45–2.05)	2.25 (1.80–2.82)	<0.001
>65	6,558	Ref.	1.19 (1.03–1.38)	1.73 (1.50–1.99)	2.38 (2.00–2.83)	<0.001
*P* for interaction			0.056	0.169	0.361	
Sex						
Male	6,933	Ref.	1.11 (0.95–1.29)	1.60 (1.38–1.86)	2.02 (1.68–2.43)	<0.001
Female	5,192	Ref.	1.35 (1.16–1.57)	1.67 (1.43–1.96)	2.55 (2.07–3.14)	<0.001
*P* for interaction			0.061	0.998	0.475	
SOFA						
≤5	6,296	Ref.	1.19 (1.03–1.37)	1.61 (1.37–1.90)	2.36 (1.84–3.03)	<0.001
>5	5,829	Ref.	1.29 (1.09–1.54)	1.86 (1.58–2.20)	2.60 (2.16–3.13)	<0.001
*P* for interaction			0.998	0.676	0.476	
CKD						
Yes	2,576	Ref.	1.31 (0.80–2.13)	1.41 (0.88–2.25)	2.22 (1.37–3.58)	<0.001
No	9,549	Ref.	1.20 (1.07–1.34)	1.65 (1.46–1.86)	2.05 (1.74–2.41)	<0.001
*P* for interaction			0.906	0.337	0.624	
Sepsis						
Yes	7,875	Ref.	1.13 (0.99–1.29)	1.51 (1.33–1.72)	2.04 (1.74–2.39)	<0.001
No	4,250	Ref.	1.31 (1.09–1.59)	1.73 (1.41–2.12)	2.50 (1.87–3.34)	<0.001
*P* for interaction			0.023	0.009	0.001	
Hypertension						
Yes	3,073	Ref.	1.23 (1.00–1.51)	1.91 (1.53–2.37)	2.92 (2.17–3.93)	<0.001
No	9,052	Ref.	1.22 (1.07–1.38)	1.55 (1.36–1.76)	2.05 (1.75–2.40)	<0.001
*P* for interaction			0.731	0.356	0.236	
DM						
Yes	1,848	Ref.	1.15 (0.82–1.61)	1.93 (1.39–2.67)	2.43 (1.66–3.57)	<0.001
No	10,277	Ref.	1.32 (1.10–1.38)	1.59 (1.41–1.78)	2.18 (1.88–2.53)	<0.001
*P* for interaction			0.774	0.197	0.859	
Malignant tumors						
Yes	2,578	Ref.	1.02 (0.84–1.24)	1.40 (1.15–1.71)	2.00 (1.56–2.57)	<0.001
No	9,547	Ref.	1.24 (1.09–1.41)	1.62 (1.42–1.85)	2.12 (1.80–2.50)	<0.001
*P* for interaction			<0.001	<0.001	<0.001	
Diuretics						
Yes	3,957	Ref.	1.18 (0.95–1.45)	1.75 (1.43–2.14)	2.26 (1.76–2.90)	<0.001
No	8,168	Ref.	1.24 (1.10–1.41)	1.57 (1.38–1.79)	2.19 (1.85–2.58)	<0.001
*P* for interaction			0.387	0.711	0.827	
Vasoactive drugs						
Yes	3,740	Ref.	1.08 (0.87–1.32)	1.49 (1.22–1.81)	2.12 (1.69–2.66)	<0.001
No	8,385	Ref.	1.22 (1.08–1.40)	1.62 (1.41–1.85)	2.06 (1.71–2.47)	<0.001
*P* for interaction			0.135	0.158	0.390	

Each stratification was adjusted for all factors of Model 3 in Table 2 except for the stratification factor itself.

### 3.6 Sensitivity analysis

The relationship between BAR and 28-day mortality and 365-day mortality remained robust when patients with missing liver function data and liver dysfunction were excluded from the complete cohort, as shown in Supplementary Table 1. In addition, the same results were observed after excluding patients with malignant tumors (Supplementary Table 2).

## 4 Discussion

AKI seriously endangers public health and life safety, and early identification of risk stratification for AKI remains an extremely challenging issue in the field (16). This study is the first to reveal that high levels of BAR are an independent risk factor for 28-day mortality and 365-day mortality in patients with AKI. This association holds even when potential confounders are taken into account. BAR predicts mortality in AKI. Comparison of AUC values showed that the accuracy of BAR was higher than that of BUN and SOFA. In addition, Kaplan–Meier survival analysis showed that AKI patients with BAR >7.14 had significantly higher 28-day mortality and 365-day mortality than patients with BAR ≤4.32. Subgroup analyses and sensitivity analysis also supported this view. Therefore, this study provides a novel, simple, and efficient biomarker for mortality risk stratification in patients with AKI.

BUN is the major end product of protein metabolism in humans. After glomerular filtration, BUN is reabsorbed in renal tubules and released in large amounts when renal perfusion is insufficient and renal function is severely impaired, which can better reflect the severity of renal injury. Furthermore, BUN can trigger immune dysfunction by promoting hypercatabolism and activating neurohumoral mechanisms, thereby driving the risk of death in patients with critical AKI (17). BUN levels have been found to be a risk factor for the need for renal replacement therapy in AKI patients (18). Furthermore, increases in BUN were associated with the outcome of the prognostic endpoints of death in AKI patients (8). However, BUN is less sensitive than glomerular filtration rate and Scr. In addition, BUN is influenced by various factors such as age, high-protein diet, gastrointestinal bleeding, dehydration, and catabolic status.

Albumin is synthesized in the liver and plays important roles, such as maintaining intravascular colloid osmotic pressure, effective circulating volume, and redox status, and is involved in molecular and drug transport (19). Existing evidence confirms that albumin exerts protective effects on the kidney due to its anti-inflammatory and antioxidant properties (20). Albumin promotes microvascular circulation and maintains renal perfusion and glomerular filtration. In addition, albumin promotes DNA synthesis in renal tubular cells through Ca^2+^-related signaling pathways and is essential for the integrity and function of proximal tubular structures (21). Low albumin, as an indicator in evaluating chronic or severe malnutrition and inflammation in humans, is generally considered to be associated with worse prognosis and outcome (22). A meta-analysis showed that hypoalbuminemia was an independent predictor of AKI development and death (23). Hypoalbuminemia makes it difficult for the body to remove toxic substances, resulting in reduced vascular volume and subsequent renal hypoperfusion, aggravating renal injury (24). In addition, albumin infusion was found to improve discharge rates and reduce 28-day mortality in septic shock and AKI patients (25). However, albumin levels are influenced by multiple factors such as liver function, catabolism, and vascular extravasation and have limited prognostic value for AKI.

BAR integrates the clinical value of BUN and albumin for AKI, taking into account hepatic and renal status, protein metabolism, and nutritional status. Theoretically, compared with BUN and albumin, it can better predict the end outcome of AKI. Studies have shown that higher BAR levels are associated with a relative lack of effective circulating blood volume, meaning that high levels of BAR may be able to evaluate renal hypoperfusion status. Currently, BAR has been shown to be associated with the risk of death from sepsis, cardiac surgery, pneumonia, chronic heart failure, and many other conditions (11, 14, 26, 27). Studies on the correlation between BAR and the risk of death from AKI are lacking. We included 12,125 patients with AKI and confirmed that initial BAR was associated with all-cause mortality in patients with AKI, regardless of whether BAR was considered a continuous or categorical variable. SOFA score is an important score for ICU patients and has a good predictive effect on mortality in critical patients (28). Notably, we found that BAR had a larger AUC than SOFA for predicting 28-day mortality and 365-day mortality, indicating that BAR has a higher predictive power for mortality outcomes in AKI than SOFA. In addition, subgroup analysis found an interaction between sepsis and malignant tumors and BAR on AKI outcomes, and BAR appeared to predict AKI mortality risk more significantly in populations with non-sepsis and non-malignancy. This may be related to the high protein catabolic rate of patients with sepsis and malignant tumors, and the degree of inflammation affecting albumin levels, which may partially interfere with the prediction of AKI outcome by BAR (29–31). However, we can see that even in AKI patients with sepsis and malignant tumors, there is an increased risk of death with increasing BAR. In conclusion, our study demonstrates a significant impact of BAR levels on prognosis in patients with AKI. Clinicians can predict the risk of death by early assessment of BAR, and those with high BAR levels should be vigilant for outcomes with poor prognosis and improve the prognosis of critical patients with AKI by active management and intervention of risk factors.

Our study remains subject to several limitations. Firstly, our study is a single-center retrospective cohort study and causality could not be determined. The prognostic mechanism of BAR on AKI requires further investigation. Potential data excursions may exist despite the application of multivariate adjustments and subgroup analyses. Further high-quality prospective multicenter studies are still needed to validate the prognostic value of BAR for all-cause mortality in AKI. Secondly, the causes of AKI vary clinically. As the causes of AKI are not recorded in detail in the MIMIC-IV database, we only performed stratified analysis for sepsis-related AKI and could not provide a comparison of the predictive value of BAR for AKI mortality with different causes. Thirdly, we investigated the relationship between initial BAR after admission and all-cause mortality, which did not allow us to assess the value of dynamic BAR. We were unable to validate the association between BAR and 365-day mortality due to the inclusion of data on only the first ICU admission and limited long-term follow-up data from the MIMIC-IV database. Fourthly, BUN levels may be associated with the patient’s diet, and we could not analyze the patient’s diet-related data using the MIMIC-IV database, which may cause some bias.

## 5 Conclusion

High BAR levels were an independent risk factor for all-cause mortality in patients with AKI, and BAR is a simple and efficient biomarker for mortality risk stratification in AKI.

## Data availability statement

The original contributions presented in the study are included in the article/Supplementary material, further inquiries can be directed to the corresponding author.

## Ethics statement

The requirement of ethical approval was waived by Massachusetts Institute of Technology and the Beth Israel Deaconess Medical Center for the studies involving humans because the database is publicly available and database loyalty identifiers are hidden to protect privacy and therefore exempt from the requirement for informed consent or ethical approval. The studies were conducted in accordance with the local legislation and institutional requirements. The ethics committee/institutional review board also waived the requirement of written informed consent for participation from the participants or the participants’ legal guardians/next of kin because the database is publicly available and database loyalty identifiers are hidden to protect privacy and therefore exempt from the requirement for informed consent or ethical approval.

## Author contributions

YS: Conceptualization, Data curation, Visualization, Writing – original draft. HD: Data curation, Visualization, Writing – original draft. JL: Formal analysis, Software, Writing – review & editing. XS: Writing – review & editing. YiZ: Writing – review & editing. QZ: Writing – review & editing. MZ: Supervision, Writing – review & editing. YuZ: Funding acquisition, Supervision, Writing – review & editing.

## Footnotes

^1^ https://mimic.mit.edu
