# Supplementary material for: Blood urea nitrogen to serum albumin ratio is associated with all-cause mortality in patients with AKI: a cohort study
Source: Front Nutr. 2024 Feb 20;11:1353956. doi: 10.3389/fnut.2024.1353956 (PMC10913022; doi:10.3389/fnut.2024.1353956)
Supplement: Supplementary file 1 [file Table_1.docx]

**TABLE S1 Sensitivity analysis after exclusion of patients with liver dysfunction and lack of liver function data**

| **Categories** | **Model 1**  **HR (95% CI)** | ***P-*value** | **Model 2**  **HR (95% CI)** | ***P-*value** | **Model 3**  **HR (95% CI)** | ***P-*value** |
| --- | --- | --- | --- | --- | --- | --- |
| 28-day mortality |  |  |  |  |  |  |
| BAR | 1.04 (1.03-1.04) | <0.001 | 1.04 (1.03-1.04) | ＜0.001 | 1.02 (1.02-0.03) | <0.001 |
| BAR (category) |  |  |  |  |  |  |
| Q1 (≤4.32) | Ref. |  | Ref. |  | Ref. |  |
| Q2 (4.32–7.14) | 1.46 (1.21-1.76) | <0.001 | 1.27 (1.05-1.54) | 0.015 | 1.03 (0.86-1.26) | 0.703 |
| Q3 (7.14–13.03) | 2.57 (2.16-3.-5) | <0.001 | 2.17 (1.82-2.60) | <0.001 | 1.57 (1.29-1.89) | <0.001 |
| Q4 (＞13.03) | 3.82 (3.23-4.51) | <0.001 | 3.32 (2.80-3.95) | <0.001 | 2.24 (1.76-2.84) | <0.001 |
| *P* for trend |  | <0.001 |  | <0.001 |  | <0.001 |
| 365-day mortality |  |  |  |  |  |  |
| BAR | 1.03 (1.03-1.04) | <0.001 | 1.03 (1.03-1.04) | <0.001 | 1.03 (1.02-1.03) | <0.001 |
| BAR (category) |  |  |  |  |  |  |
| Q1 (≤4.32) | Ref. |  | Ref. |  | Ref. |  |
| Q2 (4.32–7.14) | 1.55 (1.36-1.78) | <0.001 | 1.33 (1.16-1.52) | <0.001 | 1.16 (1.01-1.33) | 0.034 |
| Q3 (7.14–13.03) | 2.53 (2.23-2.87) | <0.001 | 2.10 (1.85-2.39) | <0.001 | 1.68 (1.46-1.93) | <0.001 |
| Q4 (＞13.03) | 3.57 (3.16-4.04) | <0.001 | 3.08 (2.71-3.49) | <0.001 | 2.41 (2.01-2.89) | <0.001 |
| *P* for trend |  | <0.001 |  | <0.001 |  | <0.001 |

Model 1 was unadjusted.

Model 2 was adjusted for sex, age, and weight.

Model 3 was adjusted for sex, age, weight, SOFA, Scr, BUN, WBC, Platelet, sepsis, hypertension, heart failure, respiratory failure, DM, and RRT.
