# Supplementary material for: Blood urea nitrogen to serum albumin ratio is associated with all-cause mortality in patients with AKI: a cohort study
Source: Front Nutr. 2024 Feb 20;11:1353956. doi: 10.3389/fnut.2024.1353956 (PMC10913022; doi:10.3389/fnut.2024.1353956)
Supplement: Supplementary file 2 [file Table_2.docx]

**TABLE S2 Sensitivity analysis after exclusion of patients with malignant tumors**

| **Categories** | **Model 1**  **HR (95% CI)** | ***P-*value** | **Model 2**  **HR (95% CI)** | ***P-*value** | **Model 3**  **HR (95% CI)** | ***P-*value** |
| --- | --- | --- | --- | --- | --- | --- |
| 28-day mortality |  |  |  |  |  |  |
| BAR | 1.04 (1.03-1.04) | <0.001 | 1.03 (1.03-1.04) | ＜0.001 | 1.01 (1.01-1.02) | <0.001 |
| BAR (category) |  |  |  |  |  |  |
| Q1 (≤4.32) | Ref. |  | Ref. |  | Ref. |  |
| Q2 (4.32–7.14) | 1.64 (1.39-1.94) | <0.001 | 1.46 (1.24-1.73) | <0.001 | 1.10 (0.93-1.31) | 0.252 |
| Q3 (7.14–13.03) | 2.79 (2.40-3.25) | <0.001 | 2.41 (2.07-2.82) | <0.001 | 1.41 (1.19-1.67) | <0.001 |
| Q4 (＞13.03) | 4.30 (3.72-4.98) | <0.001 | 3.76 (3.24-4.37) | <0.001 | 1.86 (1.52-2.29) | <0.001 |
| *P* for trend |  | <0.001 |  | <0.001 |  | <0.001 |
| 365-day mortality |  |  |  |  |  |  |
| BAR | 1.04 (1.03-1.04) | <0.001 | 1.03 (1.03-1.04) | <0.001 | 1.01 (1.01-1.02) | <0.001 |
| BAR (category) |  |  |  |  |  |  |
| Q1 (≤4.32) | Ref. |  | Ref. |  | Ref. |  |
| Q2 (4.32–7.14) | 1.78 (1.59-2.02) | <0.001 | 1.51 (1.33-1.72) | <0.001 | 1.24 (1.09-1.41) | 0.001 |
| Q3 (7.14–13.03) | 2.96 (2.63-3.33) | <0.001 | 2.44 (2.16-2.75) | <0.001 | 1.62 (1.42-1.85) | <0.001 |
| Q4 (＞13.03) | 4.37 (3.90-4.89) | <0.001 | 3.66 (3.27-4.12) | <0.001 | 2.12 (1.80-2.50) | <0.001 |
| *P* for trend |  | <0.001 |  | <0.001 |  | <0.001 |

Model 1 was unadjusted.

Model 2 was adjusted for sex, age, and weight.

Model 3 was adjusted for sex, age, weight, SOFA, Scr, BUN, WBC, Platelet, sepsis, hypertension, heart failure, respiratory failure, DM, and RRT.
